# Chicks of cavity-nesting birds do not ‘exercise’ prior to fledging

**DOI:** 10.1098/rsos.251579

**Published:** 2025-11-26

**Authors:** Kate Earle, Josh Allen, Brett Lee Hodinka, Tony Williams

**Affiliations:** ^1^ Department of Biological Sciences, Simon Fraser University, Burnaby, British Columbia, Canada

**Keywords:** fledging, exercise, development, aerobic capacity, mass loss

## Abstract

Fledging represents a key life-history transition involving a rapid increase in workload associated with a rapid transition from sedentary nestling to volant, active fledgling. Here, we tested the idea that chicks might prepare for fledging through increased voluntary activity (‘exercise’) and whether this would impact somatic and physiological development. European starling (*Sturnus vulgaris*) chicks, in cavity nests, increased levels of putative exercise (wing flapping), and more general active behaviours (e.g. perching, standing) in the five days up to fledging. However, facultative mass loss and wing growth between days 15 and 20 were independent of time spent wing flapping, standing or perching and, counterintuitively, we found a weak negative relationship between haematocrit (a measure of aerobic capacity) and time spent wing flapping or standing. Thus, although exercise is commonly associated with an increase in haematocrit in other species, this does not appear to be a mechanism for increasing pre-fledging haematocrit in chicks. Despite widespread anecdotal observations of flight preparation (e.g. wing flapping) in larger seabirds and raptors, our data suggest that exercise, or increased activity in general, does not contribute to improved development just prior to fledging: starling chicks do not ‘exercise’ enough to show somatic or physiological effects.

## 1. Introduction

In general, in humans, increased activity levels are positively correlated with physical fitness [1–3]. However, humans also *choose* to participate in ‘exercise’, i.e. planned and voluntary activity to enhance performance, reduce the risk of injury and maintain overall health. The benefits of exercise might arise through motor skill acquisition and/or the enhancement of morphological and physiological traits. Numerous laboratory studies have shown how other animals benefit from increased activity (reviewed in Yap *et al*. [4]), e.g. exhibiting increased aerobic capacity and fuel metabolism in response to exercise in wind tunnels (birds), swim tunnels (fishes) and running wheels (rodents; although markers of oxidative stress and decreased immune function also suggest costs of experimentally induced exercise in animals). Free-living animals clearly experience increased activity levels when escaping predators, migrating or foraging to meet energy demands, but these could be considered involuntary (the animal has little choice), and whether free-living animals exercise, i.e. undertake voluntary activity to improve performance, remains an open question [4,5]. Exercise-like behaviours appear more common prior to, or during, the transition from parental-dependence to independence in juveniles. Immature mammals will often engage in ‘play’ [6–9], analogous to exercise in many cases. Similarly, anecdotal evidence that chicks of open-nesting species demonstrate putative flight-preparation exercises is widespread (e.g. wing flapping: [10–12]; reviewed in [13]; see §4). However, pre-fledging exercise behaviour has seldom been quantified in birds, especially for cavity-nesting species where opportunities for exercise might be more limited owing to space constraints, and any performance benefits remain uncertain.

While ‘play’ in mammals involves interaction with the environment and conspecifics, which might promote motor skill acquisition (e.g. [14]) and have social functions (e.g. [15,16]), putative exercise behaviours in birds are performed individually and may be confined to the nest (but see [12]). Thus, it might be predicted that the function of exercise in nestling birds prior to fledging is to promote the development of traits related to flight ability. For example, many avian species demonstrate mass-overshoot recession growth profiles [17–19]. Here, nestlings achieve (and often exceed) adult mass relatively early in development, only to lose a significant portion of that mass in the approach to fledging as chicks putatively optimize their wing-loading in preparation for flight [11,20]. The observation of wing flapping and ‘push-up’ exercises in swifts approaching fledging was suggested to play a role in the observed facultative adjustment of mass recession [11], where increasing activity levels might be partly used to facilitate mass loss, as in humans. This idea has yet to be empirically tested and the mechanism by which chicks supposedly control mass recession remains unknown. Pre-fledging exercise could also promote the growth of bones and muscles that support flight [21], as suggested in burrow-nesting streaked shearwaters (*Calonectris leucomelas*, [12]), or it might accelerate physiological development [4]. The extent to which small, cavity-nesting passerine birds engage in pre-fledging exercise (cf. the widespread, but anecdotal reports in seabirds and raptors; see §4), and if this affects physiological development, remain unclear and largely undocumented. Additionally, even if exercise-like behaviour increases prior to fledging, this might simply be an artefact of social crowding effects in confined nest spaces, with activity simply being higher for chicks in larger brood sizes, rather than specific behaviour to improve performance.

Here, we used video cameras placed in nest boxes of European starlings (*Sturnus vulgaris*) to quantify putative exercise behaviour (e.g. wing flapping, wing extensions) and general activity (e.g. maintenance, sitting, standing) of nestlings approaching fledging. We measured somatic development at day 15 (asymptotic mass) and just prior to fledging at day 20, to include mass recession, and blood sampled chicks at day 20 to measure physiological development of aerobic capacity (haematocrit, haemoglobin, key components of oxygen transport; see [22–24]). We predicted that if pre-fledging activity reflected ‘exercise’ to prepare chicks for flight after fledging, then (i) activity would increase approaching fledging and (ii) activity would be independent of brood size (i.e. it does not reflect a simple social crowding effect). Further, we predicted that if increased activity levels prior to fledging are a mechanism for pre-fledging mass recession (see [25], then (iii) higher pre-fledging exercise or activity would be associated with greater mass loss. Finally, we predicted that (iv) as mean pre-fledging activity increased so too would the rapid pre-fledging development in wing growth and haematocrit, traits that remain ‘immature’, lower than adult values, at fledging (see [24]).

## 2. Methods

Field work was conducted over three breeding seasons (2021–2023) on a nest-box breeding population of European starlings at Davistead Farm, Langley, British Columbia, Canada (49°10ʹ N, 122°50ʹ W). Egg-laying was checked daily at 150 nest boxes from 1 April to determine lay date and clutch size, after which nests were monitored until fledging or failure. To observe chick activity approaching fledging, we fixed remote video cameras (Apexcam M80 Air 4K Action Camera) to the inside of nest box lids daily between days 16 and 20 (greater than 90% of chicks fledge on day 21; [26]), recording chick behaviour from directly above the nest. Fully charged cameras were placed in nest boxes between 9.00 and 15.00 h (partly to control for diurnal variation) and were left until the battery died. Mean duration of activity recordings was 62.3 and 55.8 min in 2021/2022 and 2023, respectively (before batteries died), and all activity data were standardized to 60 min for subsequent analysis. In all years, chicks were weighed (body mass, ± 0.01 g) and measured (tarsus, ± 0.01 mm; wing chord, ± 0.5 mm) on days 15 (asymptotic mass), prior to the start of video observations. Since chick tarsi are fully developed by day 15 in European starlings [23], prior to us recording activity, we did not include tarsus length in our analysis. At this time, all chicks were colour-banded and non-toxic metallic ink was used to mark the nape and/or crown of each chick, allowing individual identification of chicks from video recordings. On day 20 (approx. 1 day prior to fledging), as cameras were retrieved after video observations, chicks were weighed and measured again. Mean handling time (between initial nest disturbance and chick processing) was 4.2 ± 0.3 min in 2021/2022 and this was not related to body mass (*p* > 0.05). In 2023 only, chicks were also blood sampled at day 20, between 10.55 and 13.15 PST. Blood samples were obtained by puncturing the brachial vein with a 26.5 gauge needle and collecting blood (<1% of body mass) into heparinized capillary tubes. Haematocrit was measured with digital callipers (± 0.01 mm) following centrifugation of whole blood for 3 min at 13 000*g*). Haemoglobin concentration (g.dl^−1^ whole blood) was measured using the cyanomethaemoglobin method [27] modified for use with a microplate spectrophotometer, using 5 μl whole blood diluted in 1.25 ml Drabkin’s reagent (D5941; Sigma Aldrich Canada) with absorbance measured at 540 nm. Mean handling time before blood sampling in 2023 was 3.7 ± 0.4 min, and body mass, haematocrit and haemoglobin were all independent of handling time (*p* > 0.05 in all cases).

### 2.1. Chick behaviour

In 2021 and 2022, following preliminary analysis of observations from 2020 (J. Allen 2020, unpublished data), we focused on two putative ‘exercise’ behaviours approaching fledging: wing flapping and wing extensions, where wing extensions involved a chick extending one or both wings out more than 50% of its total wing length without subsequently flapping. For each video we recorded the total duration and total number of bouts for each of these activities for each chick per day, as well as (i) mean activity duration (daily duration h^−1^ averaged across days 16–20), and (ii) mean number of bouts (daily count h^−1^ averaged across days 16–20). Some videos were excluded from analysis (owing to males fighting, early chick fledging, and where poor lighting because of weather or chicks blocking the nest-box hole made chick identification uncertain) so final sample size was *n* = 46 chicks in *n* = 11 nests with data for 3–5 days per chick (electronic supplementary material, table S1).

In 2023, we used a more detailed ethogram to fully characterize all chick activities including time spent in the following behaviours: (i) wing flapping (as in 2021/2022)—wings fully extended and moving up and down; (ii) maintenance—self or allopreening with beak, scratching body with feet, or wiping beak; (iii) perching—using the feet to maintain an upright, balanced position at nest box entrance, not in contact with nest floor; (iv) sitting—legs tucked into body, breast in partial or full contact with nest floor; (v) standing—in an upright position with legs extended, breast not in contact with nest floor; and (vi) walking—movement consisting of small hops or steps within the nest (following [28–30]). We calculated total duration of each activity per chick per day and mean activity for all days between day 16 and 20 as above. In addition, we recorded the number of bouts (events) of partial or full extension of one wing (left or right) or both wings and summed these to get a total number of wing extension bouts comparable to 2021/2022. Sample size for activity by year, box and day are given in the electronic supplementary material, table S1. In each year some chicks fledged early (*n* = 10 in 2021, *n* = 2 in 2022, and *n* = 4 in 2023) so we did not have physiology, mass change or wing growth data for these chicks).

### 2.2. Statistical analysis

All analyses were performed in RStudio v4.2.0 (RStudio, Inc., Boston, MA, USA). We explored square-root transformation; however, results from analyses using untransformed data were qualitatively similar in all cases so we analysed and report non-transformed data (see the electronic supplementary material, table S2). We analysed pooled data for 2021/2022, and data for 2023, separately but using the same analytical methods for the different behaviours recorded in the different years (see above). We analysed variation in activity by chick age, from day 16 to day 20, using linear mixed effects models (*lmer*) with activity as the dependent variable, age as the main effect, and chick ID nested in box as a random effect (since we recorded activity from multiple chicks in the same box), with estimation of marginal means (*emmeans*). Year was included as a random effect for the pooled 2021/2022 data. In 2023 we only had data for two nest boxes from early in the pre-fledging period (days 16 and 17, when activity was generally lower; see §3) but omitting these data did not change our results so they were retained. We then calculated mean activity over days 16–20 for each chick and box, and analysed variation in activity by brood size using linear mixed effects models (*lmer*) with activity as the dependent variable, brood size as the main effect, and year (for 2021/2022 data only) and box as random effects.

We tested whether mass loss and wing growth, calculated as the difference in trait values between days 15 and 20, and haematocrit at day 20 were associated with mean activity levels over days 16–20. Haematocrit and haemoglobin were positively correlated (*r* = 0.67), so we restricted analysis to haematocrit data, and we also restricted analysis to time spent wing flapping, our putative ‘exercise’ behaviour, and the two most common ‘active’ behaviours: perching and standing. We used linear mixed effects models with the developmental or physiological trait as the dependent variable, activity as the main effect, mean chick age as a covariate (given chicks were observed on different days; electronic supplementary material, table S1) and box as a random factor.

## 3. Results

Mass at day 15 did not differ among years (*F*_2,20.5_ = 1.01 *p* = 0.38, overall mean day 15 mass = 72.3 ± 0.9 g) and mass change, between day 15 and day 20, did not differ among years (*F*_2, 24.2_, = 0.08, *p* = 0.92): 2021, −2.95 ± 0.83 g, *n* = 15; 2022, −2.87 ± 1.35 g, *n* = 19; and 2023, −1.22 ± 0.88 g, *n* = 42). Similarly, wing length at day 15 did not differ among years (*F*_2,14.0_ = 3.06, *p* = 0.08, overall mean day 15 wing length = 84.1 ± 0.7 mm), and the increase in wing length between day 15 and day 20 did not differ among years (*F*_2,18.2_ = 0.25, *p* = 0.78): 2021, +20.4 ± 0.6 mm; 2022, +19.2 ± 0.4 mm; 2023, +19.4 ± 0.3 mm.

### 3.1. Changes in activity with chick age approaching fledging

**Figure 1.**
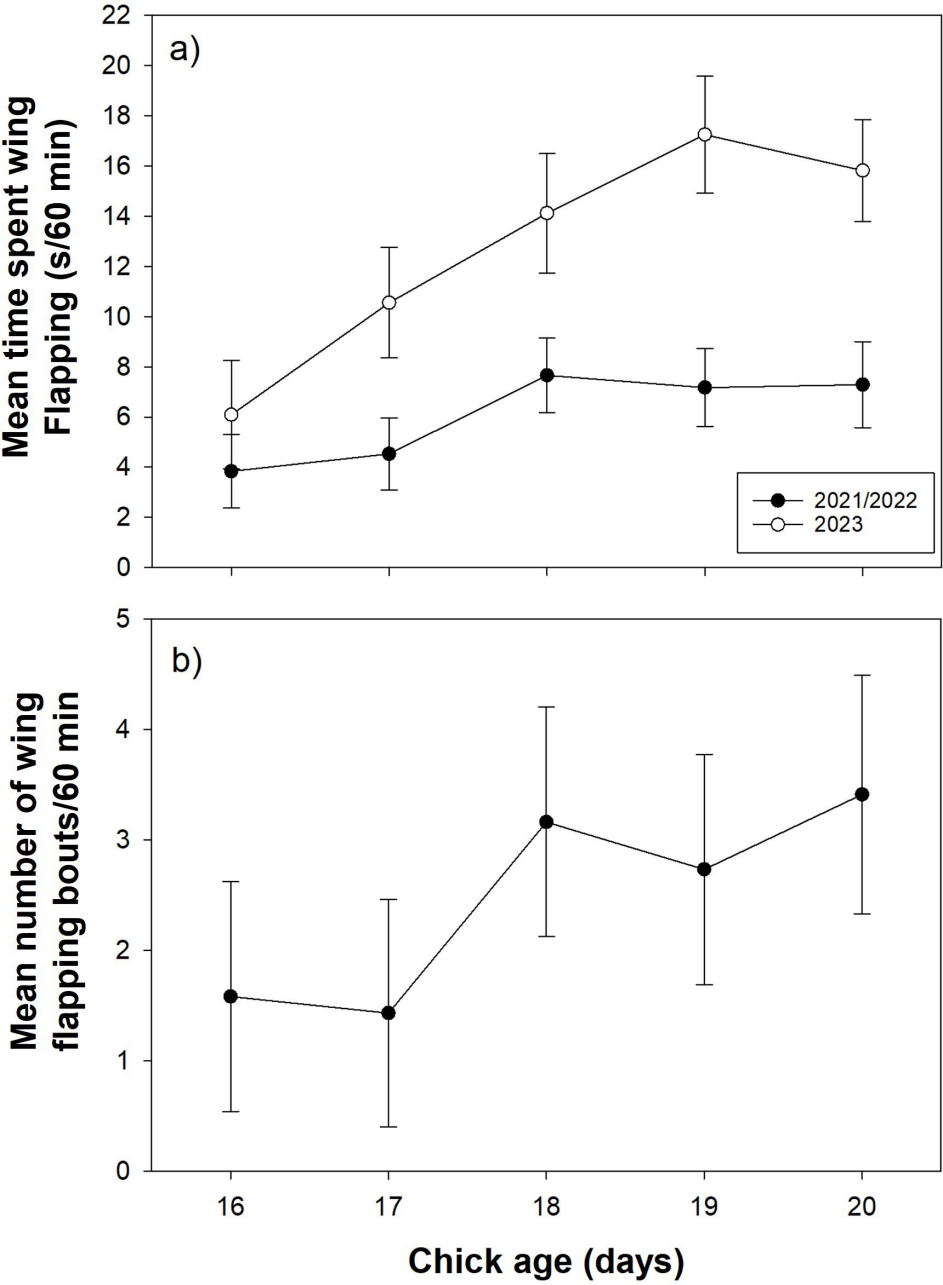
Variation in (a) mean time spent wing flapping (s h^−1^) and (b) mean number of wing flapping bouts h^−1^ (in 2021/2022 only), between day 16 and 20 prior to fledging in European starling chicks. Values are marginal means ± s.e.

For pooled 2021/2022 data, both time spent wing flapping (*F*_4,141.0_ = 3.63, *p* = 0.008; figure 1a) and number of wing flapping bouts (*F*_4,134.9_ = 6.33, *p* < 0.001; figure 1b) increased with chick age between days 16 and 20. Chicks spent more time wing flapping (*p* = 0.05) and engaged in more wing flapping bouts (*p* < 0.025) at days 18, 19 and 20 compared to day 16. However, chicks only spent an average of 7.3 ± 1.7 s h^−1^ wing flapping, across 3.4 ± 1.1 bouts, at day 20 (electronic supplementary material, table S2). There was no overall main effect of age on time spent in wing extensions (*F*_4,136.6_ = 1.44, *p* = 0.225), with no difference between day 16 and 20 (estimate −0.33 ± 2.14, *p* = 0.88; electronic supplementary material, table S3). Similarly, there was no main effect of age on number of wing extension bouts (*F*_4,141.8_ = 2.15, *p* = 0.078) and no difference between day 16 and 20 (estimate −0.43 ± 0.70, *p* = 0.538; electronic supplementary material, table S3).

**Figure 2.**
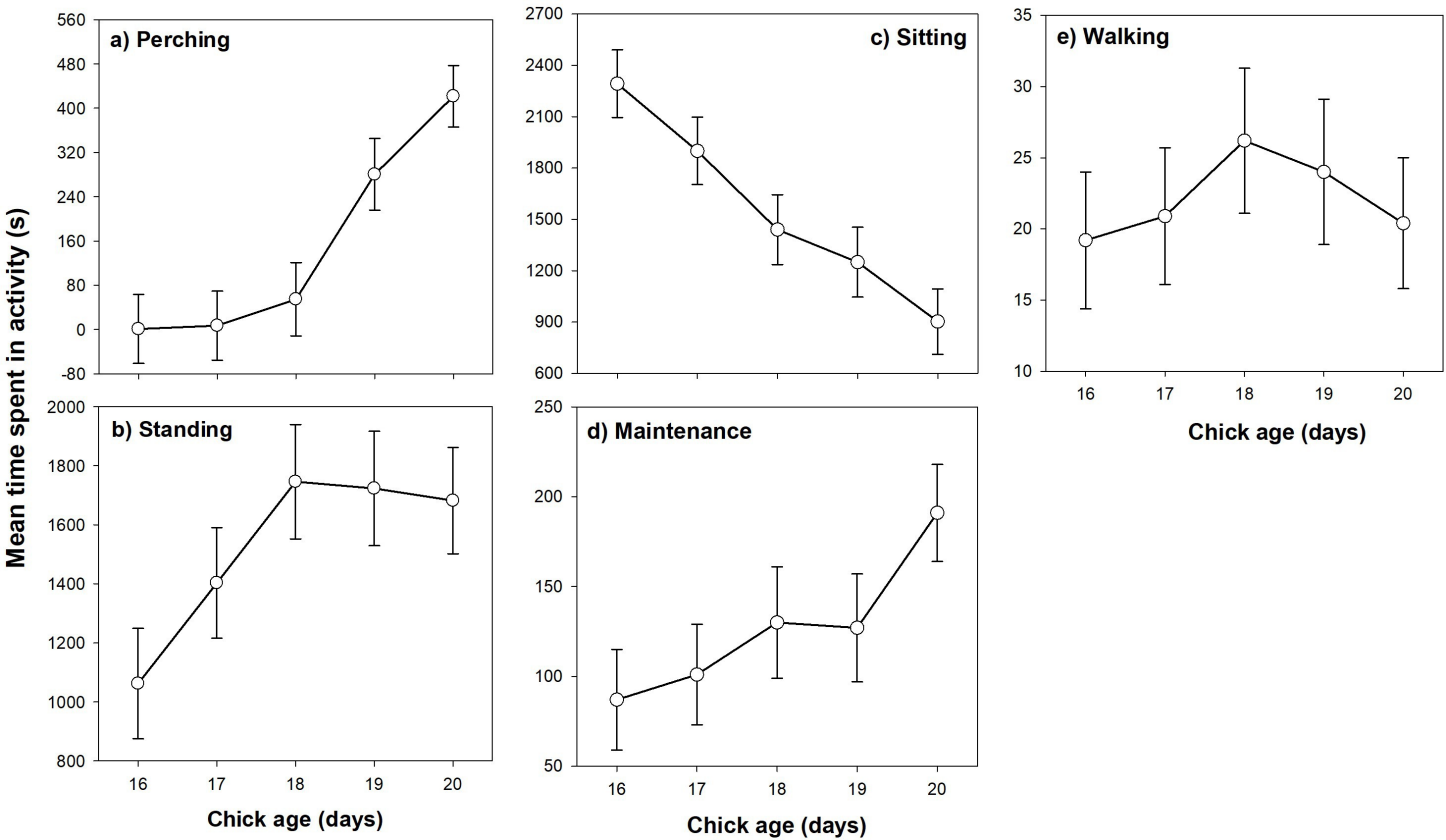
Variation in mean time spent, (a) perching, (b) standing, (c) sitting, (d) in maintenance, and (e) walking (s h^−1^), between day 16 and 20 prior to fledging in European starling chicks. Values are marginal means ± s.e. for 2023 data.

In 2023, time spent wing flapping increased between days 16 and 20 (*F*_4,101.3_ = 4.55, *p* = 0.002) and chicks spent more time wing flapping at day 20 (15.8 ± 2.0 s h^−1^) compared to day 16 (6.1 ± 2.2 s, *t* = 3.38, *p* = 0.009; figure 1a). Chicks markedly increased time in ‘active’ behaviours approaching fledging: perching (*F*_4,107.6_ = 11.26, *p* < 0.001; figure 2a) and standing (*F*_4,133.7_ = 4.64.1, *p* = 0.002; figure 2b), and decreased time in ‘passive’ behaviours: sitting (*F*_4,103.9_ = 16.4, *p* < 0.001; figure 2c). Change in time spent in maintenance activity with age was marginally non-significant (*F*_4,138_ = 2.26, *p* = 0.077; figure 2d) while time spent walking (*F*_4,134.9_ = 0.55, *p* = 0.70; figure 2e) and number of wing extension bouts (*F*_4,78.6_ = 1.24, *p* = 0.30; see the electronic supplementary material, table S2) did not change with age. Overall, at day 20 just prior to fledging, chicks spent most time standing (1682 s, 52.0% of total observed time) and sitting (903 s, 27.9%), less time perching (421 s, 13.1%) and in maintenance (191.0 s, 5.9%), and very little time wing flapping (15.8 s, 0.5%) or walking (20.4 s, 0.6%).

In 2021/2022, mean time spent in the four measured activities (wing flapping duration and bouts, wing extension duration and bouts) between days 16 and 20 were all independent of brood size (*p* > 0.25 in all cases; electronic supplementary material, table S4). Similarly, in 2023 mean time spent in wing flapping, maintenance, perching and sitting was independent of brood size (*p* ≥ 0.10 in all cases), but there was a marginally non-significant effect of brood size on time spent standing (*F*_1,7.2_ = 5.19, *p* = 0.056) and time spent walking (*F*_1,7.09_ = 4.58, *p* = 0.07; electronic supplementary material, table S4) with chicks in the largest broods of six being more active.

### 3.2. Pre-fledging activity and somatic and physiological development

**Figure 3.**
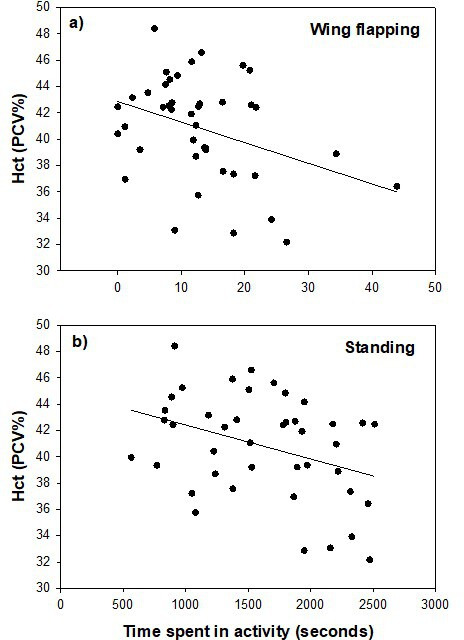
Relationship between pre-fledging haematocrit (packed cell volume, PCV %) on day 20 and mean time per chick spent (a) wing flapping and (b) standing (s h^−1^) over days 16–20 (2023 data only).

Using pooled data for all years, both mass change (*F*_1,69.7_ = 0.02, *p* = 0.90, estimate 0.009 ± 0.072) and wing growth (*F*_1,72.5_ = 0.56, *p* = 0.46, estimate 0.023 ± 0.031; electronic supplementary material, table S5) between days 16 and 20 were independent of mean time spent wing flapping. Mass change was independent of mean time spent standing (*F*_1,38.1_ = 1.15, *p* = 0.29, estimate 0.002 ± 0.001) and mean time spent perching (*F*_1,39.0_ = 0.55, *p* = 0.46, estimate −0.002 ± 0.003). Similarly, wing growth was independent of time spent standing (*F*_1,38.7_ = 1.62, *p* = 0.21, estimate 0.001 ± 0.006) and perching (*F*_1,38.8_ = 3.01, *p* = 0.091, estimate −0.002 ± 0.001). By contrast, haematocrit on day 20 was negatively related both to mean time spent flapping (*F*_1,38.9_ = 8.06, *p* = 0.007; estimate −0.175 ± 0.062; figure 3a) and mean time spent standing (*F*_1,39_ = 4.28, *p* = 0.045, estimate −0.002 ± 0.001; figure 3b) but was independent of time spent perching (*F*_1,38.7_ = 0.034, *p* = 0.85, estimate −0.001 ± 0.003; electronic supplementary material, table S5).

## 4. Discussion

Fledging represents a key life-history transition involving a rapid increase in workload, i.e. onset of active flight, associated with a rapid transition from sedentary nestling to volant, active fledgling [26]. In many avian species, the immediate post-fledging period is marked by high mortality: 50% or greater during the first 3–4 weeks post-fledging, with predation accounting for 55%–80% of fledgling mortality [31,32]. Poor flight performance of newly fledged juveniles, i.e. an inability to evade predators, is thought to explain this high mortality and is determined, in part, by developmental maturity at fledging (e.g. the morphological or physiological capacity for sustained flight [33]). In humans, participants in ‘Couch to 5K’ programmes do not simply get off the couch and immediately run 5 km, rather they ramp up training, or voluntary activity, over 6-9 weeks anticipating the increase in workload. It seems intuitive that free-living birds might, similarly, prepare for workload transitions such as at fledging, or the transition from relatively sedentary incubation to active chick rearing in breeding adults. However, little is known about ‘exercise’ in other species [4,34–36].

Consistent with this general hypothesis, in European starling chicks in cavity nests, both specific activity such as wing flapping, and more general active behaviours (e.g. perching, standing) did increase in the five days leading up to fledging whereas other passive behaviours either did not change (maintenance, walking) or decreased (sitting). These changes in activity were not simply a side-effect of social crowding or interactions among chicks within broods, since brood size explained little or no variation in activity. Although by day 20 chicks spent approximately 52% of their time standing in the nest, wing flapping was very rare, accounting for only approximately 0.5% of total observation time. We predicted that (i) mass change would increase with greater activity, if this provided a mechanism for facultative mass recession [11], and (ii) that activity would be positively related to pre-fledging wing growth and aerobic physiology [24] if it contributed to increased flight capability. We found no evidence to support either of these ideas: mass change and wing growth between days 15 and 20 were independent of mean time spent wing flapping, standing or perching. This does not support the prediction that increased activity levels are a mechanism for mass recession [5] but rather, the mechanism for mass recession may be related to physiological processes such as the metabolization of triglycerides [23] or water loss in maturing tissues [37,38]. We did not observe our birds doing ‘push-ups’ as observed in common swifts (*Apus apus*), potentially as a way to assess body mass or wing-loading to determine their ‘required’ pre-fledging mass recession and optimize post-fledging flight ability [11,39]. Although chicks could simply assess body mass by standing, via mechanical pathways involving mechanosensory cells and peripheral proprioceptors in legs muscles [30], we found no relationship between mass loss and time spent standing.

Counterintuitively, we found a weak *negative* relationship between haematocrit and time spent wing flapping or standing. Even though European starling chicks reach asymptotic mass and skeletal size equivalent to, or greater than, that of adults prior to fledging (approx. day 15 post-hatch) they are physiologically immature at fledging with haematocrit and haemoglobin values approximately 78% of adult values [26]. In adults of several species, exercise or increased activity is associated with an increase in haematocrit ([40]; reviewed in [4]) but this does not appear to be a mechanism for increasing pre-fledging haematocrit in chicks. This supports the idea that physiological development of haematocrit and haemoglobin, components of aerobic capacity are constrained or canalized [24,41] even though there is evidence that this physiological immaturity at fledging contributes to variation in flight ability post-fledging [26,42] and that this has direct fitness consequences in terms of recruitment [43].

Anecdotally, it is widely assumed that chicks *do* practise flight prior to fledging, though observations mainly come from large birds with open nests such as albatrosses and raptors (reviewed in [13]) For example, albatrosses (*Diomedia* spp.) ‘*give his* [sic] *wings a workout as fledging approaches*’, flapping their wings and even ‘hovering’ at high wind speeds ‘*as a way to strengthen their flight muscles*’ (https://www.allaboutbirds.org/cams/fledge-watch-albatross-chicks-begin-flight-preparations/). Similarly, ‘young [osprey, *Pandion haliaetus*] often exercise their wings, hovering over the nest’ [44]. Chicks of burrow-nesting seabirds repeatedly emerge from their nests in the late chick-rearing period [10] and are thought to use these ‘excursions’ to exercise their wings [12]. Nevertheless, empirical evidence that these activities do improve muscle or physiological function is largely lacking. In streaked shearwater (*C. leucomelas*), Yoda *et al*. [12] showed that wing-length growth rate and body mass were positively correlated with mean excursion time although they acknowledged that this could reflect the fact that chicks with more advanced development conducted longer excursions, rather than longer excursions accelerating development. Our data suggest that exercise, or increased activity in general, does not contribute to improved or accelerated somatic or physiological development just prior to fledging: if European starling chicks do ‘exercise’ (e.g., wing flapping) they do not exercise enough to show physiological effects. This is consistent with the few studies that have prevented, or constrained, wing flapping and shown no effects on fledging, indicating that wing movement is not necessary for postnatal development of basic wing-flapping and flight [45,46]. This makes the workload transition that chicks face at fledging even more remarkable.

## Data Availability

The dataset generated and analysed during this study is available in the Dryad Data Repository [47]. Supplementary material is available online [48].
